# Complete genome sequences of 20 *Streptococcus pyogenes* clinical isolates from patients with invasive infections in Japan

**DOI:** 10.1128/mra.00165-26

**Published:** 2026-07-20

**Authors:** Koushin Kuroki, Masaki Yamada, Masanori Hashino, Kazuhiro Horiba

**Affiliations:** 1 Center for Postgraduate Education and Training, National Center for Child Health and Development, Tokyo, Japan; 2 Division of Infectious Diseases, Department of Medical Subspecialties, National Center for Child Health and Development, Tokyo, Japan; 3 Laboratory of Microbial Genomics, Pathogen Genomics Center, National Institute of Infectious Diseases, Japan Institute for Health Security, Tokyo, Japan; University of Pittsburgh School of Medicine, Pittsburgh, Pennsylvania, USA

**Keywords:** *Streptococcus pyogenes*, group A streptococcus, invasive group A streptococcal infection

## Abstract

We report the complete genome sequences of 20 clinical *Streptococcus pyogenes* isolates obtained from patients with invasive infections in Japan. These genome sequences are provided as a resource to support molecular epidemiology and pathogenicity studies of invasive *S. pyogenes* infections.

## ANNOUNCEMENT

*Streptococcus pyogenes* (group A Streptococcus [GAS]) is an aerobic gram-positive coccus that causes a spectrum of diseases from pharyngitis to invasive infections. Invasive GAS infection is defined by the presence of GAS at a normally sterile site (1). GAS infection spread globally from 2022 to 2023 and also spread in Japan from 2023 to 2024 (1, 2). The *emm* genotyping is used for strain classification, and several *emm* types are associated with the development of invasive GAS infections (3). Here, we report the 21 complete genome sequences of GAS, which were isolated from invasive infections at a single medical center for children in Japan, between 2019 and 2025.

From 2019 to 2024, *S. pyogenes* isolated from various specimens of cases with streptococcal infections via aseptic puncture or tissue incision at the National Center for Child Health and Development were stored at −80°C. Subsequently, stocked *S. pyogenes* were cultured overnight on blood agar at 35°C in a 5% CO₂ atmosphere. A single colony obtained from this culture was then streaked onto a separate blood agar plate and further cultured under the same conditions as a monoculture. The patients consisted of pediatric cases with a median age of 4.5 years (range, 0–14 years), and 11 patients were male. Single colonies were collected and resuspended in 400 µL phosphate-buffered saline and 400 µL phenol/chloroform, followed by bead-beating for 10 min in ZR BasingBead Lysis Tubes (Zymoresearch, Irvine, CA, USA). After centrifugation at 15,000 rpm for 5 min, the supernatant was subjected to genomic DNA purification by MinElute PCR Purification Kit (Qiagen, Hilden, Germany). DNA quantity and purity were assessed using the Qubit fluorescence assay system (Invitrogen, Carlsbad, CA, USA) and TapeStation System (Agilent Technologies, Santa Clara, CA, USA). Sequencing libraries were prepared according to manufacturer instructions: for short-read sequencing using the QIAseq FX DNA Library Kit (Qiagen) and for long-read sequencing using the Native Barcoding Kit 96 (SQK-NBD112.96; Oxford Nanopore Technologies, Oxford, UK). Illumina sequencing was performed using the NextSeq 1000 System (Illumina, San Diego, CA, USA) with a 2 × 151 bp paired-end protocol. Nanopore sequencing was performed using the GridION platform (Oxford Nanopore Technologies) with R10.4.0 flow cells (FRO-MIN114). For nanopore sequencing, MinKNOW (version 22.12.5) and Guppy (version 6.4.6) (Oxford Nanopore Technologies) were used for raw data base calling and adapter trimming. The Illumina data were preprocessed using Fastp (version 0.23.2) to remove adapters and low-quality sequences and NanoFilt (version 2.8.0) for Nanopore sequencing data (4, 5). Unicycler (v0.5.1) was used in a hybrid assembly with Illumina data and the GridION data (6). The complete genome sequence was then annotated, and taxonomy was identified using DFAST version 1.6.0 with default parameters (7). DFAST is a web-based service that supports bacterial genome annotation, taxonomy identification, and preparation of submission files for the DDBJ, DNA Data Bank of Japan, as a public database (https://dfast.ddbj.nig.ac.jp/). The M type (emm type) was determined by GAS-J, a web-based tool for emm typing of *Streptococcus pyogenes* based on sequence analysis (8).

Assembly sizes ranged from 1,785,063 to 1,937,180 bp with a GC content of 38.31%–38.59%. The other assembly statistics and general genome information are summarized in Table 1. This genomic data could support future research investigating the pathogenic mechanisms of GAS in different disease types, as well as epidemiological studies.

**TABLE 1:** Assembly statistics and general genome information for *S. pyogenes* strains

Strain^*a*^	Symptom^*b*^	Year of collection	M type^*c*^	Nextseq output (reads)	GridION output (reads)	GridION read N50 (bases)	Coverage (fold)	Assembly results	Genome size (bp)	GC (%)	Accession no.
GAS5	Arthritis	2019	1	5,837,080	478,645	8,035	935.5	Complete	1,785,063	38.57	AP045146
GAS6	Bacteremia	2020	28	1,424,309	43,327	6,290	229.7	Complete	1,847,352	38.31	AP045147
GAS7	Bacteremia	2020	3.93	3,469,605	863,066	5,541	531.1	Complete	1,900,590	38.59	AP045148
GAS8	Bacteremia	2020	12	6,012,483	1,207,650	6,241	829.2	Complete	1,845,928	38.57	AP045149
GAS9	Bacteremia	2022	77	4,084,347	642,714	6,477	621.5	Complete	1,902,255	38.46	AP045150
GAS10	Bacteremia	2023	89	3,078,712	15,148	7,017	492.9	Complete	1,824,830	38.51	AP045151
GAS11	Bacteremia	2023	12	3,818,667	240,346	5,767	626.6	Complete	1,795,653	38.55	AP045152
GAS12	Bacteremia	2023	1	5,279,489	101,791	5,551	809.3	Complete	1,879,296	38.54	AP045153
GAS13	Arthritis	2023	2	3,329,671	38,409	6,271	496.0	Complete	1,937,180	38.40	AP045154
GAS16	Bacteremia	2023	1	4,274,129	108,879	7,085	673.1	Complete	1,837,180	38.53	AP045155
GAS17	Bacteremia	2023	12	4,143,473	47,859	7,250	676.1	Complete	1,797,126	38.55	AP045156
GAS18	Bacteremia	2024	1	2,817,641	40,871	6,334	447.6	Complete	1,835,818	38.54	AP045157
GAS19	Bacteremia	2024	12	4,033,094	864,858	6,407	650.7	Complete	1,796,683	38.55	AP045158
GAS21	Bacteremia	2024	12	3,808,051	120,023	4,921	614.6	Complete	1,795,893	38.55	AP045160
GAS22	Bacteremia	2024	1	3,910,585	24,845	5,826	605.4	complete	1,879,295	38.54	AP045161
GAS23	Arthritis	2024	1	4,655,863	118,089	6,420	733.9	Complete	1,835,966	38.54	AP045162
GAS24	Bacteremia	2024	1	4,503,705	542,031	5,706	704.7	Complete	1,838,596	38.53	AP045163
GAS25	Empyema	2024	12	4,153,766	22,823	6,046	664.8	Complete	1,831,939	38.50	AP045164
GAS26	Bacteremia	2024	12	4,011,758	43,287	6,107	647.4	Complete	1,795,548	38.51	AP045165
GAS27	Osteomyelitis	2024	89	4,331,700	70,882	6,715	696.2	Complete	1,791,977	38.55	AP045166

^*a*^ Strain identifiers were abbreviated (e.g., NCCHD_GAS_5 was denoted as GAS5).

^*b*^ Symptom refers to the clinical diagnosis.

^*c*^ M type (emm type) was determined by emm genotyping for strain classification.

## Data Availability

The complete genome sequences of the 20 *S. pyogenes* strains in this study have been deposited in DDBJ under the accession numbers shown in Table 1. The raw Illumina and GridION read data can be found in the National Center for Biotechnology Information Sequence Read Archive under the BioProject accession number PRJDB37658.
